# *In vitro* skin anti-aging potential evaluation of methanolic extract of aerial parts from *Acalypha indica Linn*

**DOI:** 10.6026/973206300211796

**Published:** 2025-07-31

**Authors:** Rajashree Sriram, G. Prakash Yoganandam

**Affiliations:** 1 Department of Pharmacognosy, Mother Theresa Post Graduate & Research Institute of Health Sciences (Government of Puducherry Institution), Affiliated to Pondicherry University, Puducherry, India

**Keywords:** Skin aging, treatment, aerial parts, MAIE, collagen, biosynthesis

## Abstract

The methanolic extract of aerial parts from *Acalypha indica* (MAIE) as potential candidates for skin aging treatment is of interest. The skin antiaging property was evaluated by its antioxidant and extracellular matrix degrading enzyme-inhibiting activity. The study reported the highest total phenolic content and total flavonoid content with notable antioxidant activity (ABTS and DPPH assay) at the concentration of 1, 2 and 5 µg/ml. The inhibitory potential of MAIE against Elastase (IC50 = 21.79 ± 0.01µg/ml) and MMP-1 (36.29 ± 0.01µg/ml), the key enzymes linked to acceleration of the aging process explicit enzyme inhibition. *In vitro* studies with L929 cells demonstrated that MAIE was noncytotoxic up to of 5 µg/mL, significantly reduced cell viability of irradiated cell by 48.33%, indicating its potent photoprotective capacity and exhibited effective Collagen biosynthesis by Sirius Red method. MAIE can be further developed as a natural skin antiaging agent in the cosmetics, cosmeceutical and pharmaceutical industries.

## Background:

A complex multifactorial biological process occurring in all living beings is Skin aging, driven by a blend of endogenous or intrinsic aging and endogenous or extrinsic aging determinants [1]. The inevitable intrinsic aging, determined primarily by a person's underlying genetics, while the evitable extrinsic / photoaging, determined by environmental stressors such as exposure to pollutants, lifestyle, use of alcohol, smoking, *etc.*, in turn, regulates how the preset frame of opportunity is exploited in course of individual trajectory. However, the traits of an aging (intrinsic and extrinsic) skin can be identified by their distinct features such as dryness, wrinkles, susceptibility to bruising, elasticity loss and mottled dyspigmentation [2]. Skin vitality and aesthetics are considered as key indicators of overall and health and wellness. Consequently, the demand for skin rejuvenation strategies has surged with a worldwide annual expenditure expected to grow from $US24.6 billion to around $US44.5 billion by 2030 [3]. Despite of the skin's protective function, the UV (ultraviolet) radiation has a significant impact on most cells, including keratinocytes, the outer layer of the skin. UV radiation can have deleterious effect on skin resulting in photoaging, wrinkles and hyperpigmentation, which is considered to be one of the most censorious risk factors [4]. In particular, UVB is the most precarious environmental carcinogen known with regard to human health through production of reactive oxygen species (ROS) and through the activation of ROS-generating enzyme. The uncontrolled collection of ROS in cells including Keratinocytes end up in oxidative alterations of lipids, proteins, nucleic acids and other intracellular molecules that eventually result in activation of complex signaling pathways, followed by matrix metalloproteinases (MMP's) induction in cutaneous cells [5] and resulting in COL breakdown. Also, the COL produced through TGF-β/Smad signaling pathway is downregulated. Further, during aging process, the tissue inhibitors of metalloproteinases (TIMP's) are downregulated, thus the presence of senescent cells supports ECM (Extracellular matrix) degradation by advancing chronic inflammatory responses and COL breakdown [6]. Thus in photoaging, there is depletion of COL production in a vicious cycle and the comprehensive drop in the COL content results from an upsurge in COL deterioration as well as fall in COL synthesis. The generated ROS leads to a rise of MMP's expression and TGF-β signaling inhibition, subsequently causing COL breakdown and its reduced biosynthesis, prevents fibroblasts and ECM from interacting mechanically [7]. Telangiectasia, or noticeable blood vessels, results from UV damage causing small vessels to dilate, is a condition that typically appears on the face that worsens with prolonged sun exposure. Photoaging profoundly affects skin health, extending beyond aesthetics to heightened cancer susceptibility. Identifying its indicators enables early intervention, enhancing skin condition and minimizing UV-induced DNA damage that elevates cancer risk [8]. Bio prospecting active natural products for the prevention and treatment of skin disorders, specifically skin aging processes, plant species are also found to be an extraordinary source of raw materials for the development of standardized herbal-derived products based with scientific evaluations of their efficacy, safety and quality control [9]. *Acalypha indica* (Family: Euphorbiaceae) a common weed, annual herb of 30 -70 cm height, found distributed in India, Indian Ocean islands, South-East Asia, Oceania, East Africa to Southern Africa including South Africa and introduced into warmer parts of the world. In some practices in India, the whole plant instead of leaves is used to treat bronchitis, where the whole plant is crushed for fresh juice [10]. *A. indica* has been identified to be used in the Indian traditional system of medicine such as Ayurveda and Siddha. It has been found to possess anti-venom, anti-inflammatory, anti-oxidative properties, as anti-psoriasis agent and is also reported to be used in ailments related to skin [11, 12- 13]. Studies have identified saponins, tannins, flavonoids, essential oils, steroids, triterpenoids, ascorbic acid, β-sitosterol, kaempferol and Quercetin (geroprotective in premature aging) in *A. indica* [14-15]. However, compared with long history of its applications, there is no report on the effect of *A. indica* extract against skin aging studies. Therefore, it is of interest to investigate the *in vitro* skin anti-aging potential efficiency and cytotoxicity of the aerial parts of *A. indica* in order to evaluate its potential application in cosmetic and cosmeceutical applications.

## Materials and Methods:

## Preparation and extraction:

100gm of shade dried aerial parts of the plant were minced, weighed and powdered. The powdered drug was macerated for 24 hours each time with 80% methanol at room temperature (3X4L). The filtrate obtained was gathered and evaporated under reduced pressure in a rotary evaporator at 40°C and the percentage yield of MAIE was calculated.

## Determination of total phenolics:

In accordance with Folin-Ciocalteu's method, the assay was performed to evaluate the phenolic content of the test samples, as previously described by Kurt-Celep *et al.* [16] Freshly diluted sample solutions (20µl) were mixed with 75µl Na2Co3 (20%w/v) and with 100 µl of Folin-Ciocalteu reagent then diluted with water (1:9). The solution was then incubated for 30 min at 45°C, the absorbance of the mixture was then recorded spectrophotometrically at 765nm. Results were expressed as mg of gallic acid equivalents (GAE) per g of extract.

## Determination of total flavonoids:

The determination of total flavonoid content of the fractions by the modified aluminium chloride method as described previously by Chutoprapat *et al.* (2020) [17]. 50 µl of MAIE, 25 µl of 10% Al Cl3 freshly prepared and 100 µl of 5% NaNo2 were and added 25 µl of NaOH and the reaction was then allowed followed by 10 min of incubation of the mixtures at room temperature was performed. After incubation, the absorbances at the wavelength of 450 nm were measured by microplate reader. The flavonoid contents were asserted as mg quercetin equivalents (QE) in 1g of extract.

## ABTS radical scavenging activity:

The antioxidant activity was investigated by the ABTS Free Radical scavenging Assay (Pientaweeratch *et al.* 2016) [18] compared with L-(+)-ascorbic acid as positive control. The ABTS reagent was prepared by reacting 7 mM ABTS solution with 140 mM potassium persulfate (88µL) at room temperature for 12-16 h, until the reaction was completed and the absorbance was stabled. After which it was diluted with distilled water (1:49 v/v). To determine the antioxidant activity, (20 µL) the diluted samples and (180 µL) ABTS.+ solution was deposited in the wells of a 96-well plate, with the determinations carried out in 3 replicates and the reaction proceeded for 6 min in the dark and the absorbance was measured at 734 nm. The ability to scavenge ABTS radical was calculated using the formula,

ABTS % Scavenging = [(A_0_- A_1_)/ A_0_] X 100

Where,

A_0_ is the control absorbance

A_1_ is the samples absorbance

IC_50_ values of the results were calculated from the graph plotted between % inhibition and the sample concentrations.

## Determination of 2, 2- Diphenyl-1-picrylhydrazyl (DPPH) free radical scavenging activity:

The free radical scavenging activity were determined by the 2,2- Diphenyl-1-picrylhydrazyl (DPPH) method as followed by Boonpisuttinant *et al.* (2023) [19]. A combination of 100 µl of the MAIE extract or L- ascorbic acid (Sigma-Aldrich, USA) as positive control and 100 µl of 0.1 mg/ml of ethanolic DPPH (Sigma-Aldrich, USA) solution were added to a 96 well microplate. The mixture was then incubated at room temperature in a dark place for 30 min and the absorbances were measured at 515 nm wavelength using a microplate reader and the % of scavenging activity is calculated using the same formula as used above.

## Elastase inhibition assay:

The anti-elastase activity was carried out using 0.2 mM tris-HCL buffer solution (pH 8.0). A stock solution of porcine pancreatic elastase (P.E., E.C. 3.4.21.36) was prepared with distilled water of 3.33 mg/mL concentration. The substrate N-Succinyl-Ala-Ala-ρ-Nitroanilide (AAAPVN) was dissolved in buffer (1.6 mM). Before the addition of the substrate, the MAIE was incubated with P.E (1 µg/mL) for 15 min at 37°C. At the end of 15 min pre-incubation, to the mixture of enzyme and 1 mg/ml MAIE, 0.8mM AAAPVN substrate was added and again subjected to incubation for 15 min at 37°C. An amount of 0.25 mg/mL EGCG was used a positive control. Following the incubation periods, the rate of inhibition was measured by Microplate Spectrophotometer at 410 nm wavelength [20].

## MMP-1 inhibitory assay:

The *in vitro* collagenase inhibitions focusing on MMP-1 was determined according to Maiti *et al.* by diluting 1 µL inhibitor N-Isobutyl-N-(4- methoxyphenylsulfonyl)-glycylhydroxamic acid (NNGH) in 200 µL assay buffer consisting of 50 mmol/L 4-(2-Hydroxyethyl)- 1-piperazineethanesulfonic acid, 10 mmol/L C_a_C_l2_, 0.05% Brij-35 and 1 mmol/L 5, 5'-dithiobis (2-nitrobenzoic acid). Then, as needed, the substrate was diluted in assay buffer, to make up a volume of 10 µL per well followed by addition of MMP-1 (fibroblasts collagenase) enzyme diluted in assay buffer to a volume of 20 µL per well. Before the assay was performed, the mixture was subjected to heating to room temperature. In to each well plate of the microplate, the blank assay buffer (90 µL), control assay buffer (without inhibitor) (70 µL) and NNGH inhibitor assay buffer (50 µL) were pipetted and allowed to equilibrate to assay temperature at 37 °C. To each well (except blank), a volume of 20 µL MMP-1 were added. To this NNGH inhibitors (20 µL) were added to the inhibitor NNGH wells and subjected to 30 min incubation at 37 °C. To each well 10 µL of substrate was added and the plate was read continuously for 10 min at 1 min interval at 412 nm [21]. In this study EGCG was used as a standard. The collagenase inhibition activities were finally expressed as an IC_50_ value.

## Cell cultures:

L929 mouse fibroblasts cell line was maintained in Dulbecco's modified Eagle's medium CDMEM; Invitrogen) (SIGMA- Aldrich) supplemented with 1 X Antibiotic antimycotic solution and 10% Fetal bovine serum (Hi media, India) under the standard conditions at 37C under the 5%CO_2_ incubator for 24 hours before experiments.

## MTT assay:

The various concentrations of MAIE were investigated for the cells cytotoxicity using 3-(4,5-dimethylthiazol-2-yl)-2,5-diphenylte trazolium bromide (MTT) assay based on the method described by Boonpisuttinant *et al.* 2023 with slight modifications [19]. The mouse fibroblasts at the density of 1 X 104 cells/ well plates, adjusted the volume to 180 µL with the DMEM, incubated at 37 °C under a 5% CO2 atmosphere for 24h. With different concentrations of the extract (2.5 -100µg/ml) were then added and incubated under the same conditions for 24h. Then the cells were subjected to washing thrice with 10mM PBS at pH 6.8 and added 1 ml of 0.5 mg/ml of MTT solution (SIGMA-Aldrich). After incubation at room temperature for 3h MTT solution was removed and to each well 100µl of Dimethyl Sulfoxide (DMSO) was added. The plates were then shaken at 200 rpm for 15 min and the absorbances of the solutions at 570nm wavelength were measured using a microplate reader with the cell viability calculated according to the following formula,

%Cell viability = [A _sample_ / A _control_] X 100

Where A _control_ is the absorbance of the Control and A sample is the absorbance of the samples.

## Ultraviolet irradiation and Phototoxicity measurement:

The phototoxicity measurement study was based on the method described by Kong *et al.* 2019 with slight modifications [22]. Cells were UV B treated using two fluorescent Philips lamps (280- 370 nm) and measured by UV B radiometer with sensor. The cells were cultivated in a culture dish until to being confluency of approximately 80%. After the of medium, the cells were rinsed by PBS. The cells were pretreated with or without MAIE and exposed to a dose of 30J/cm^2^ radiations, followed by washing with PBS once and irradiated under a thin layer of PBS, followed by immediate incubation with the drug or free media for 24h. The control samples were treated without exposing to UVB lamps and the photoprotection of MAIE was calculated.

## Collagen biosynthesis using Sirius red staining assay:

The collagen biosynthesis of the *Acalypha indica* extract on L929 mouse fibroblasts as the method described by Százc *et al.* (2023) [23]. The L929 mouse fibroblasts at a density of 5 X 10^4^ cell/well seeded into a 24 well plate in DMEM media with 1X Antibiotic Antimycotic solution and 10% Fetal bovine serum (FBS) and incubated at 37 °C and incubated under a 5%Co2 for 24h, followed by replacement of medium containing 0% FBS or 1mM ascorbate and test agents for 48h. After that the medium was aspirated and the cells were washed with 500 µL/well PBS. Subsequently, followed by cell fixation with 200 µL/well Kahle's solution at room temperature for 15 min and again washing with PBS (500 µL/well) and finally stained with Sirius red solution (200 µL/well) at room temperature for 1h. The cells were then washed with 400 µL/well HcL solution for 2 times and the images were captured using an inverted phase contrast microscope (optiKa SRL, (Model: IM- 37L4)) using 10X eyepiece and 10X objective lens. The cell bound Sirius red were eluted with 100 µL/well of 0.1 M NaOH solution and the absorbance of the colour intensity at the wavelength of 540 nm were measured using a microplate reader using blanks. The percentage of the Collagen amount was calculated by using the following formula:

% Collagen content = (C_sample_/ C_control_) X 100

Where C_sample_ was the collagen content of the sample and

C_control_ was the collagen content of the control

## Statistical analysis:

The data obtained were expressed as mean ± S.D. Results were statistically analysed by Microsoft Excel and one-way analysis of variance (ANOVA) using Graph Pad Prism version 10.4.1.

## Results and Discussion:

The % yield of MAIE was found to be 20.13% w/w and the extract was used for further study. The ability to donate hydrogen or electrons and also to form stable radical intermediates, makes the phenolic compounds to act as antioxidants [24]. In this study, the total phenolic content of MAIE was studied using the Folin-Ciocalteu method. The total content of phenolic compound was estimated using Gallic acid as a standard solution. Upon addition of the Folin-Ciocalteu reagent, the maximum absorption of Gallic acid at 765nm wavelength was studied. Before the estimation of total phenolic content, the standard calibration curve of gallic acid was estimated at different concentrations of 1, 2.5, 5, 10, 25 µg/ml and the absorbance were measured respectively. Then total phenolic content MAIE was calculated using the regression equation y= mx+c. The analysis of the study was repeated thrice and the mean value of the absorbance was measured. Determining the concentration of the sample solution using the calibration curve of the sample absorbance, the study reported the highest total phenolic content of 72.5 GAE/g, thus indicating its highest presence of bio-actives responsible for its antioxidant activity. The total flavonoid content (TFC) was measured by modified aluminium chloride calorimetric assay and MAIE prepared by maceration extraction technique has exhibited a flavonoid content of 24.77 QE/g extract. Notably, the significant antioxidant properties correspond to the presence of Phenolic and Flavonoid compounds [18]. The present study of MAIE has reported a high content of TPC and TFC, thus making it as a potential candidate for many bioactivities such as antioxidant and its anti-microbial contents.

MAIE demonstrated the ABTS and DPPH free radical scavenging effect in a dose dependent manner with similar order. The ABTS free radical scavenging effect of MAIE at concentration of 1, 2 and 5 µg/ml was found to be 20.99 %, 28.27% and 45.7% respectively and the values of the control Ascorbic acid was found to be 59.56 %, 86.5% and 97.45% respectively (Figure 1a - see PDF). Similarly, the DPPH free radical scavenging effect of MAIE at concentration of 1, 2 and 5 µg/ml were evaluated and the determination of the reaction kinetic types DPPH-H is a product of the reaction between DPPH. and an antioxidant (AH) as shown in the following equation:

DPPH. + AH ↔ DPPH-H + A. -------------------------------- (Equation 1)

However, the reaction reversibility is evaluated by adding DPPH-H at the end of the reaction. Thus, by performing the DPPH assay, the ability of the extract to act as a donor of hydrogen atoms or electrons in transforming of DPPH. into its reduced form DPPH-H was investigated, where the examined sample can reduce the stable, purple-colored radical DPPH into yellow-colored DPPH-H [25]. The values of the extract at the concentration of 1, 2 and 5 µg/ml were reported to be 23.63%, 26.15% and 50.92% respectively and the values of the control Ascorbic acid were found to be 48.56 %, 79.56% and 90.12% respectively. (Figure 1b - see PDF) Together these results indicate the MAIE has a very good antioxidant activity but lower when compared to that of the control compound L-Ascorbic acid.

The activities of elastase and MMP-1 inhibitions of MAIE were studied and compared using the standard EGCG. Both assays were studied at 5, 10, 15, 25, 50 and 100 µg/ml concentration and MAIE inhibited both collagenase and elastase in a dose-dependent manner. EGCG was used as a positive control due to its effective protease inhibition capacity, [26] positive inhibition of collagenase enzyme, expression of mRNA stromelysin induced by IL-1β activities and protection from skin damage caused by UV rays [21]. MAIE exhibited anti-elastase activity with an inhibitory concentration (IC50) of 21.79 ± 0.01 µg/ml (Figure 2a - see PDF) and that of EGCG was found to be 12.73 ± 0.002 µg/ml (Figure 2b - see PDF). From the study, it can be reported that MAIE can be a good elastase inhibitor, as the inhibitory concentration of the extract though was not higher than the standard, but exhibits comparable anti-elastase activity to the inhibitory concentration of EGCG. Alongside, MAIE exhibited a different profile for anti-collagenase activity when compared to that of standard EGCG. MAIE exhibited anti-MMP-1 activity with an inhibitory concentration (IC_50_) of 36.29 ± 0.01 µg/ml (Figure 3a - see PDF) and that of EGCG was found to be 43.48 ± 0.010 µg/ml (Figure 3b - see PDF), thus it is notable that the extract expressed a relatively high anti-collagenase activity than EGCG.

The cytotoxicity of MAIE was studied using L929 mouse fibroblasts by the MTT assay and the cells were treated with the extract at the concentrations ranging from 1-50 µg/mL. The study reported that MAIE at the concentration of 1, 2.5 and 5 µg/mL had no cytotoxicity on L929 mouse fibroblasts, as they gave > 80% relative viability compared to the untreated groups. Despite this, the higher concentration of the MAIE was considered cytotoxic on these cells. The study exhibited an IC_50_ of 10.73 ± 0.05 µg/ml (Figure 4 - see PDF). The L929 cells treated with MAIE in the MTT assay, did not cause any significant decrease in cell viability at a concentration below 5 µg/mL, so we chose 1, 2.5 and 5 µg/mL concentration, for later experiments. As shown in the Figure 5 (see PDF), the photoprotective effects of MAIE on L929 cells under UVB irradiation of 30 mJ cm-2 for 24 hrs were evident. The results expressed that the cell viability of irradiated cell was significantly reduced by 48.33% (p <0.0001) compared with control cells and the data showed MAIE at 5 µg/mL increased cell viability about 11.50% respectively, indicating that MAIE possessed increased UV protection.

The COL in L929 mouse fibroblasts cells were visualized using Sirius Red following the standard histological procedure and then microscopic images were taken using bright-field and polarized light illumination techniques (Figure 6a - see PDF). The MAIE at the concentration of 1, 2.5, 5 µg/mL concentration showed the stimulation of collagen biosynthesis on L929 mouse fibroblasts cells determined by Sirius Red method. The concentration of 5 µg/mL of MAIE and L-ascorbic acid was determined to be the highest concentration that showed no cytotoxicity on the L929 mouse fibroblasts and the deposition of red stain indicates the presence of COL produced within cells. The intensity of the colour produced is evident in the positive control (AA: Ascorbic acid 5 µg/mL). The results obtained in this study were represented as Mean ± SEM **** p < 0.0001, by One-way ANNOVA test (Figure 6b - see PDF). MAIE at the concentration of 5 µg/mL can be considered as the most effective concentration that acts as a photoprotective, enhances COL production, thus can be considered as a potential candidate for skin antiaging formulations. Further, the present study found that methanolic extraction of aerial parts possesses high antioxidant activity, supports fibroblasts viability and enhances COL synthesis. The antioxidant activities of MAIE was evidenced by using ABTS radical cation scavenging assay and by DPPH scavenging method, which were widely used in plant and food research for screening antioxidant activity [19]. From the study reported by Sanseera *et al.* 2012, on hexane, chloroform and methanolic extracts to investigate the antioxidant activities of aerial parts of *A. indica*, the plant was reported to possess a rich source of antioxidant activity, thus indicating its effectiveness in treating diseases caused by overproduction of radicals [25]. The antioxidant activity of *A. indica* as reported by Nahrstedt *et al.* 2006 attributes to the presence of Kaempferol glycosides such as mauritianin, clitorin, nicotiflorin and biorobin from *A. indica*. Further the presence of other phytochemicals such as flavonoids, tannins, cynogentic glucoside, acalyphin, acalyphamide, aurantiamide, succinimide and the pyranoquinolinone alkaloid flindersin were confirmed in this plant [27] Another study reported by Ibrahim *et al.* 2021, evidenced the exhibition of higher antioxidant activity of aerial parts of *A. indica* compared to the root, due to the presence of phytochemicals especially phenolic compounds and also reported its low cytotoxicity towards fibroblasts and its capacity to stimulate fibroblasts viability [28]. Notably, an UV irradiated skin, exhibits a decrease in the TGF-β expression, which in turn brings down an altered COL production and enhances elastin production, thus causing changes in skin structure [29]. According to the histological and ultrastructural studies of aged skin, it has been reported that the major alterations are found to be localized in the dermal ECM [30]. In numerous physiological and pathological events, it is observed that TGFβ Signaling plays critical contextual-specific roles. Premature production of COL in a photoaged skin could be connected to the activation of TGFβ /SMAD3 Signaling in the dermis, [31] thus in order to aim for a possible photoaging treatment, it is significant that we understand the pleiotrophic effects of TGFβ. And one such medicinal plant that is acclaimed traditionally to treat skin related ailments is *A. indica* [32]. As a traditional practice followed by Asians especially in India and Nepal, *A. indica* is used to treat several diseases such as rheumatoid arthritis, wound healing and skin irritation.

The plant is also used to treat insect bites to reduce the skin soreness and the leaves were used as good antibacterial, good anti-fungal and are also found to possess good antioxidants that help protecting the skin from external hazards Though *A. indica* has been reported to have high moisture content up to 80% ideal for body hydration, [10] and has been reported to upregulate TGF-β1, elevated COL synthesis on topical application [11] there are no studies documented on its skin anti-aging effect. It is essential to emphasize that the screening outcomes serve as the initial indication of antioxidant and anti-aging properties. Nonetheless, to propose it as a potential candidate for cosmeceutical utilization, additional research is imperative.

## Conclusion:

The potential of MAIE against skin anti-aging is reported. The extract showed relevant *in vitro* antioxidant activity. It also exhibited excellent inhibitory potential against elastase and collagenase enzymes, demonstrated notable UV protection. MAIE showed relevant COL biosynthesis stimulation which is beneficial for procollagen synthesis and rearrangement of damaged skin. These findings indicate MAIE as a potential skin anti-aging candidate and the results correlated well with the practise of using the extract traditionally for skin related ailments.

## Disclosure statement:

The authors declare no conflicts of interest.

## Ethical approval:

This article does not contain any human participant and animal work.

## Author contributions:

All the authors contributed equally to this work.

## Funding:

Self-funding.

## Data availability statement:

All dataset supporting this article is available within the article and its supplementary files.
